# Kinetin stimulates differentiation of C2C12 myoblasts

**DOI:** 10.1371/journal.pone.0258419

**Published:** 2021-10-13

**Authors:** Michal Mielcarek, Mark Isalan

**Affiliations:** 1 Department of Life Sciences, Imperial College London, London, United Kingdom; 2 Imperial College Centre for Synthetic Biology, Imperial College London, London, United Kingdom; Waterford Institute of Technology, IRELAND

## Abstract

Kinetin or N6-furfuryladenine (K) belongs to a class of plant hormones called cytokinins, which are biologically active molecules modulating many aspects of plant growth and development. However, biological activities of cytokinins are not only limited to plants; their effects on animals have been widely reported in the literature. Here, we found that Kinetin is a potent small molecule that efficiently stimulates differentiation of C2C12 myoblasts into myotubes *in vitro*. The highest efficacy was achieved at 1μM and 10μM Kinetin concentrations, in both mitogen-poor and rich media. More importantly, Kinetin was able to strongly stimulate the MyoD-dependent conversion of fibroblasts into myotubes. Kinetin alone did not give rise to fibroblast conversion and required MyoD; this demonstrates that Kinetin augments the molecular repertoire of necessary key regulatory factors to facilitate MyoD-mediated myogenic differentiation. This novel Kinetin pro-myogenic function may be explained by its ability to alter intracellular calcium levels and by its potential to impact on Reactive Oxygen Species (ROS) signalling. Taken together, our findings unravel the effects of a new class of small molecules with potent pro-myogenic activities. This opens up new therapeutic avenues with potential for treating skeletal muscle diseases related to muscle aging and wasting.

## Introduction

Kinetin or N6-furfuryladenine (K) belongs to a class of plant hormones called cytokinins, which are both adenine and non-adenine derived molecules [1]. Initially, Kinetin was isolated from autoclaved herring sperm DNA and was shown to be a potent activator of the proliferation of cultured tobacco pith cells [2]. At the time, there was a consensus that Kinetin did not occur naturally. However, later studies identified Kinetin in extracts from root nodules of *Casuarina equisetifolia*, infected by the bacterium *Frankia* [3], and in coconut water [4]. Although Kinetin was the first such small molecule to be identified, cytokinins consist of a number of small molecules that are structurally-similar to Kinetin, where position N6 of an adenine ring is substituted with either a benzyl residue (N6-benzyladenine, BA), a hydroxylated isopentenyl residue (*trans* and *cis* Zeatin, Zea), or an isopentenyl residue (N6-(Δ^2^-isopentenyl) adenine, iP). Zeatin was initially identified as the first naturally-occurring cytokinin, in immature maize endosperm [5]. Later, it was confirmed that this cytokinin occurs as two isomers: *trans*, which is active in all plant species, and *cis*, which is present in all plants but active only in a subset [6]. It has been widely described that cytokinins are biologically active molecules modulating many aspects of plant growth and development, including a broad spectrum of biological processes like cell division, shoot initiation and growth, senescence, nutrient uptake and phyllotaxis. Cytokinins are also involved in vascular, gametophyte, and embryonic development, as well as in the response to biotic and abiotic factors (reviewed in [1, 7]).

The biological activities of cytokinins are not only limited to plants and their effects on other organisms, including animals, have been reported widely in the literature. Two species of fruit flies, *Zaprionus paravittiger* and *Zaprionus indianus*, showed prolonged lifespans after treatment with Kinetin [8], likely by enhancing catalase activity [9]. A similar effect has been observed when studying nematode worms. For example, Kinetin treatment (200μM) significantly increased longevity in *Caenorhabditis elegans*, and treated groups showed a greater resistance to oxidative and heat stress [10]. The anti-aging properties of Kinetin have been also documented in human mammary skin fibroblasts. When supplemented with 40–200μM Kinetin, these cells displayed a reduction in a number of morphological features linked to aging, such as improved cell size and shape, reduced autofluorescence and improved cytoskeleton appearance. In addition, Kinetin treatment improved cytokinesis and reduced the number of cells with multiple nuclei [11]. Another study, performed on 3D-reconstructed skin, showed a beneficial effect of Kinetin via increased levels of proliferation and differentiation markers [12].

The anti-aging properties of Kinetin can be partially explained by its ability to protect DNA against oxidative damage mediated by the Fenton reaction [13]. Interestingly, elevated levels of Kinetin have been found in the urine of cancer patients and it has been suggested that the presence of Kinetin might indicate the presence of increased oxidative stress and DNA damage [14]. On the other hand, Kinetin and other cytokinins were shown to modulate a number of processes, enzymes and transcription factors related to damage-protection in mammals. For instance, Kinetin induces activity of superoxide dismutase (SOD), catalase (CTL) and glutathione peroxidase (GP) in the human promyelocytic cell line HL-60 [15]. In the mouse hippocampal cell line HT22, Kinetin modulates translocation of the transcription factor Nuclear factor erythroid 2–related factor 2 (Nrf2), which is a major regulator of xenobiotic metabolism. Nrf2 thus translocates to the nucleus to reach its target, heme oxygenase-1 (HO-1) [16]. Kinetin treatment of late-passage endothelial cells leads to altered expression levels of proteins involved in cytoskeletal function, intracellular trafficking, cell-cycle progression, translation, protein turnover, coagulation and collagen maintenance [17]. Finally, Isopentenyladenine (iP) has been shown to bind the gamma subunit of AMP-activated protein kinase (AMPK) and function as its activator [18].

Taken together, cytokinins have been shown to regulate vital biological processes, not only in plants but also in mammalian cells, including those identified elsewhere to be critical for skeletal muscle differentiation and regeneration, like reactive oxygen signalling and oxidative homeostasis [19]. Hence, in the current study we sought to link these ideas and investigate the effects of Kinetin and two other cytokinins (namely N6-benzyladenine and trans-Zeatin), on the differentiation process of myoblasts into myotubes *in vitro*. For this purpose, we utilised the well-described murine C2C12 cell line, which is a gold standard to study muscle differentiation *in vitro*, when grown under mitogen-poor conditions, and which is unable to differentiate in mitogen-rich media. To monitor the differentiation process, we used well-established markers like Myogenin and Muscle Creatine Kinase (MCK) activity. Any link between Kinetin and muscle differentiation could potentially lead to new small molecule treatments to intervene in diseases of muscle degeneration and aging.

## Material and methods

### RNA extraction and Taqman real-time PCR expression analysis

Cells were briefly washed with PBS, followed by extraction with TRizol (Ambion, UK). The whole cell suspension was transferred to Epperdorf tubes, followed by a freezing-thawing cycle. Total RNA from cell lines was extracted with a mini-RNA kit, according to manufacturer instructions (Qiagen). The reverse transcription reaction (RT) was performed using MMLV superscript reverse transcriptase (Invitrogen) and random hexamers (Operon), as described previously [20]. The final RT reaction was diluted 10-fold in nuclease-free water (Sigma) for further Taqman-qPCR reactions. All Taqman-qPCR reactions were performed as described previously [21], using a Cycler 480 Real Time Thermal Block Cycler (Roche). Estimation of mRNA copy number was determined in triplicate for each RNA sample, by comparison to the geometric mean of three endogenous housekeeping genes, *Hprt*, *Gapdh* and *18S*, as described previously [22]. The Myogenin and MyoD Taqman assays were purchased from ABI as described previously [23].

### Cell culture

C3H10T1/2 (ATCC CCL-226) and C2C12 (ATCC CRL-1772) cell lines were grown in D-MEM (1,000 mg/ml glucose) medium containing 15% or 10% FCS, 100 U/ml of penicillin, 100 μg/ml of streptomycin and 0.292 mg/ml L-glutamine (Life Technologies, UK), called hereafter Growth Medium (GM). Cells were grown for 2–3 days to reach 60–80% confluence, in a humidified atmosphere containing 5% CO_2_, at 37° C. Cells were detached from dishes with trypsin solution (0.05% trypsin-EDTA) (Life Technologies, UK) and split in a ratio of 1 to 6, as previously described [24]. For experimental procedures, cells were counted using a cell haemocytometer and seeded in 6-well plates, at 10^5^ cells per well density. All drugs used in this study including Kinetin (K), Kinetin riboside (KR), N6-benzyladenine (BA), trans-zeatin (ZEA) were at analytical grade, purchased from Sigma Aldrich. All cytokinins were dissolved in DMSO (Sigma Aldrich), prepared freshly ahead of cell culture tests. DMSO was used as a negative control in all experimental settings. Typically, when used, all cytokinins were added to the medium the next day (within 12–18 hours) after seeding cells. The experimental design workflow has been presented as S1 Fig.

### Differentiation of C2C12 myoblasts into myotubes

To differentiate C2C12 cell lines to myotubes, cells were grown in Differentiate Medium (DM) containing D-MEM (1,000 mg/ml glucose) supplemented with 2% (v/v) Horse Serum (HS), 100 U/ml of penicillin, 100 μg/ml of streptomycin and 0.292 mg/ml L-glutamine. Cells were cultured in DM medium up to 96 hours after removal of mitotic stimuli, until myotubes were formed. Medium was replaced daily and fresh medium was applied.

### MyoD-dependent conversion of fibroblast cell lines

C3H10T1/2 fibroblasts are also able to form myotubes in a so-called conversion assay, as described previously [24]. To convert fibroblasts into myotubes, cells were transiently transfected with the pCMV-MyoD plasmid (Addgene 8398; a gift from Andrew Lassar) [25], using Lipofectamine 2000 reagent and Opti-Mem medium (Life Technologies, UK), accordingly to the manufacturer’s instructions (Life Technologies, UK). 24 hours after transfection, medium was replaced with DM supplemented with 5% Horse Serum, 100 U/ml of penicillin, 100 μg/ml of streptomycin and 0.292 mg/ml L-glutamine. Cytokinins or DMSO were added 24 hours post transfection. Medium was replaced daily with fresh medium. Typically, the conversion of fibroblasts into myotubes was terminated 6 days after transfection, and cells were harvested for further biochemical assays.

### Cytotoxicity assay

Cell death was assessed using an MTT (3-(4,5-dimethylthiazol-2-yl)-2,5-diphenyltetrazolium bromide) Assay kit (Abcam), according to the manufacturer’s instructions. Absorbance was read at 590nm using a Microplate Reader (BioRad).

### Muscle Creatine Kinase activity assay

The activity of the Muscle Creatine Kinase was measured using Creatine Kinase assay kit accordingly to the manufacturer’s instructions (Sigma Aldrich). Absorbance was read at 340nm using a Microplate Reader (BioRad). Briefly, cells were washed twice with ice-cold PBS and scraped. Cells were centrifuged at 1000 × *g* and resuspended in PBS containing 0.1% Tween-20, and incubated on ice for 30 min. The lysates were centrifuged at 10,000 × *g* for 15 min at 4°C, and the protein concentration in the supernatant was determined using a Pierce protein assay kit. Typically, 100μl of sample was used for creatine kinase assay.

### Determination of ROS levels

Reactive oxygen species (ROS) were measured using a DCFDA / H2DCFDA—Cellular ROS Assay Kit (Abcam; ab113851), according to the manufacturer’s instructions. Briefly, C2C12 myoblasts were seeded in 96-well plates in growth media (GM) at cell density of 5000 cells per well and incubated overnight as described above. Next, adhered cells were treated with Kinetin at different concentrations. ROS levels were measured at 12h and 24h after Kinetin was added and cells were stained with DCFDA for 45 min. At least 10 biological replicates per each condition were performed. Fluorescence was determined using a Microplate Reader (BioRad) at excitation/emission wavelengths of 485 and 535 nm, respectively.

### Statistical analysis

All data were analysed with SPSS (IBM) and One-way ANOVA with a Bonferroni *post-hoc* test. Each experiment was performed with at least 3 biological replicates.

## Results

**Fig 1:**
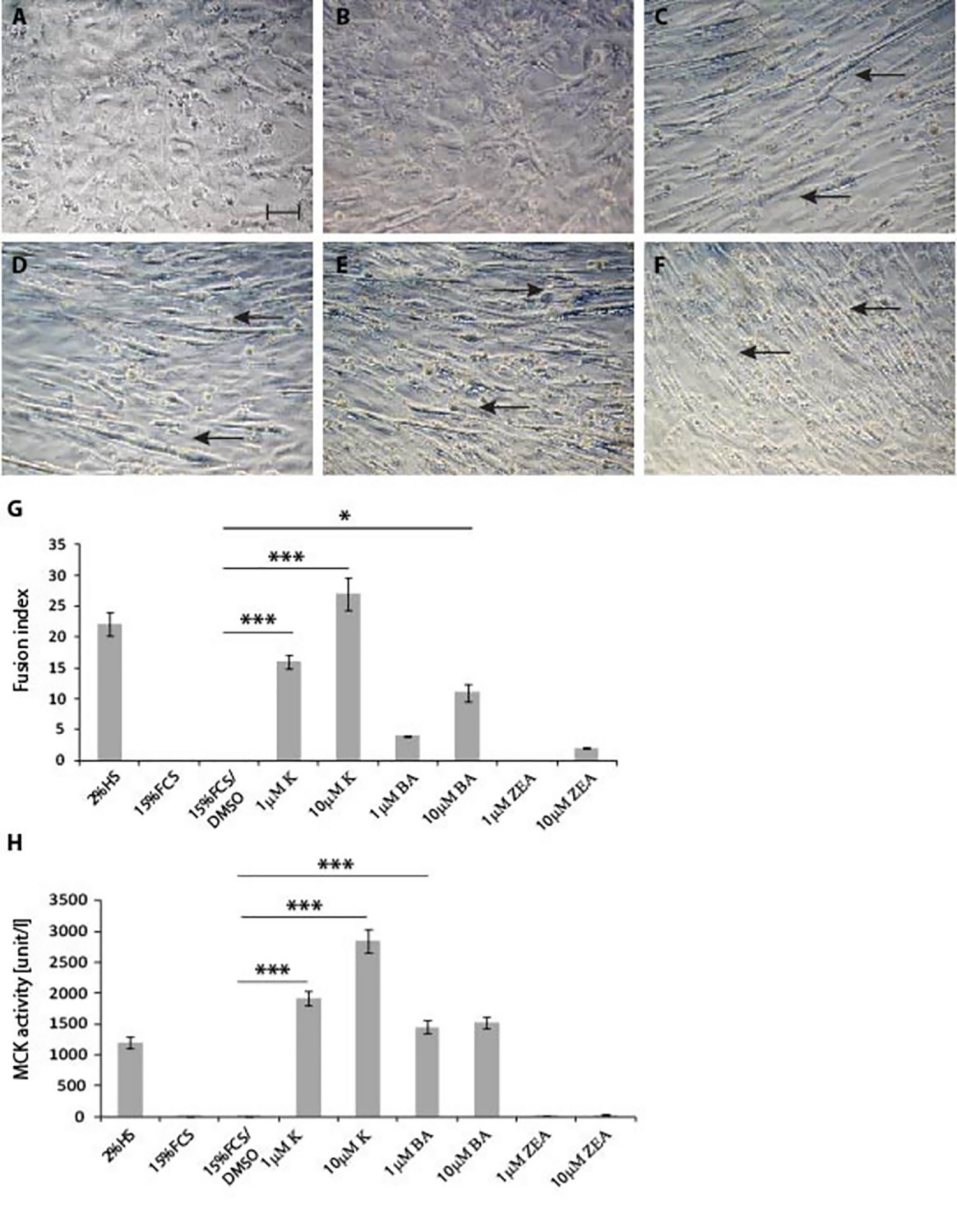
Effect of cytokinins on differentiation of C2C12 cells. Representative pictograms of C2C12 myoblasts grown in GM growth medium supplemented with 15% FCS for 7 days, in the presence of **A)** DMSO, **B)** 10μM trans-Zeatin, **C)** 1μM Kinetin, **D)** 10μM Kinetin, **E)** 1μM N6-benzyladenine, **F)** 10μM N6-benzyladenine. **G)** Fusion index was significantly higher when C2C12 cells were grown in GM in the presence of Kinetin, at 1μM and 10μM concentrations, in comparison to cells grown in the presence of GM and DMSO. Similarly, 1μM and 10μM BA increased the Fusion index in comparison to cells grown in the presence of GM and DMSO. As a positive control, C2C12 cells were grown in DM supplemented with 2%HS for 4 days. **H)** Kinetin at 1μM and 10μM concentration, and N6-benzyladenine at 1μM and 10μM concentration, significantly induced MCK activity in comparison to cells grown in DM and DMSO. Error bars are ± SEM (n = 9). One-way Anova with Bonferroni *post hoc* test: *p<0.05, **p<0.01, ***p<0.001. Black arrows indicate apparent presence of myotubes. Scale bar 50μm. FCS (Foetal Calf Serum); HS (Horse Serum); DM (Differentiation medium); GM (Growth medium); K (Kinetin); BA (N6-benzyladenine); ZEA (trans-Zeatin); MCK (Muscle Creatine Kinase).

To investigate the potential effects of Kinetin and other cytokinins on muscle differentiation, we used a well-established cellular model: the differentiation of C2C12 myoblasts into myotubes. This is based on the fact that C2C12 myoblasts undergo a differentiation process under mitogen-poor conditions. These conditions are typically achieved by replacing mitogen-rich growth medium (GM, e.g. 10–15% FCS) with a mitogen-deprived differentiation medium (DM, e.g. 2 or 5% HS) [26]. In order to sensitively monitor any potential effect of cytokinins on myoblasts, we initially used strongly mitogen-rich medium (15% FCS). Since the differentiation process is significantly inhibited here, differentiation is delayed in time and can take up to 14 days [27]. Hence, we cultured C2C12 cells in GM supplemented with the following cytokinins: trans-Zeatin, Kinetin and N6-benzyladenine, at 1μM and 10μM concentrations. All treatments were in comparison to C2C12 myoblasts supplemented with DMSO only (negative control; Fig 1A).

First, there was a significant enhancement effect on the differentiation of myoblasts with Kinetin, after 7 days in culture (Fig 1C and 1D). N6-benzyladenine also enhanced C2C12 differentiation (Fig 1E and 1F), at both 1 μM and 10 μM concentrations. By contrast, trans-Zeatin did not show any effect even at the highest 10 μM concentration (Fig 1B). Next, we quantified the effect of cytokinins on myoblast differentiation using a Fusion index (Fig 1G). We found that Kinetin had the largest effect on the formation of myotubes, at both tested concentrations. N6-benzyladenine also showed a statistically significant effect but only at the highest 10 μM concentration. Zeatin supplementation did not show an increase in the Fusion index, in comparison to cells treated with DMSO only (Fig 1G). Notably, we used C2C12 cells grown in DM (2%HS) as a positive control. Under these conditions, only Kinetin addition reaches and surpasses the level of myotube formation achieved by the DM positive control.

We further quantified the pro-differentiation effect of cytokinins using an unbiased assay based on muscle creatine kinase activity (MCK), which increases during myoblast differentiation. We found a significant increase of MCK activity in the presence of both Kinetin and N6-benzyladenine, at 1μM and 10μM concentrations, whereas trans-Zeatin did not activate MCK in comparison to myoblasts supplemented with DMSO (Fig 1H).

**Fig 2:**
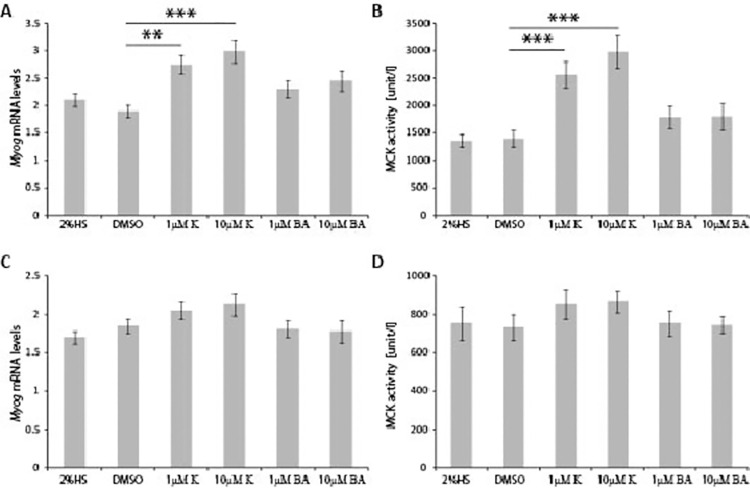
Kinetin but not N6-benzyladenine (BA) increases C2C12 differentiation in the DM differentiation medium. C2C12 myoblasts were grown in DM supplemented with 2% horse serum in the presence of DMSO, 1μM or 10μM Kinetin, 1μM or 10μM BA. Kinetin but not BA increases **A)** *Myog* mRNA levels and **B)** MCK activity, 3 days post induction of myoblast differentiation. This effect was diminished 6 days post induction of myoblast differentiation, when **C)** *Myog* mRNA levels and **D)** MCK activity were not elevated in the presence of either Kinetin or N6-benzyladenine. Error bars are ± SEM (n = 6). One-way Anova with Bonferroni *post hoc* test: *p<0.05, **p<0.01, ***p<0.001. HS (Horse Serum); DM (Differentiation Medium); K (Kinetin); BA (N6-benzyladenine); MCK (Muscle Creatine Kinase); *Myog* (Myogenin).

We next assessed the effect of Kinetin and N6-benzyladenine on the C2C12 myoblasts, when grown in the pro-myotube differentiation medium (2%HS). Here, Kinetin (but not N6-benzyladenine) significantly enhanced differentiation of myoblasts at 3 days post induction, in the mitogen-poor medium, based on an analysis of *Myog* mRNA levels (a marker of myotube formation; Fig 2A) and MCK activity (Fig 2B). These treatments were in comparison to cells grown in DM supplemented with DMSO-only as a negative control. These effects diminished 6 days post removal of mitogen, when we no longer found increased levels of either *Myog* transcripts (Fig 2C) or MCK activity (Fig 2D). This result may indicate that Kinetin enhances the differentiation of myoblasts during its early phase. Since there was no extra effect of N6-benzyladenine on the C2C12 myoblasts grown in the DM, we performed further experiments using Kinetin only as our lead compound: it enhances myoblast differentiation, both in the growth medium and in differentiation medium.

**Fig 3:**
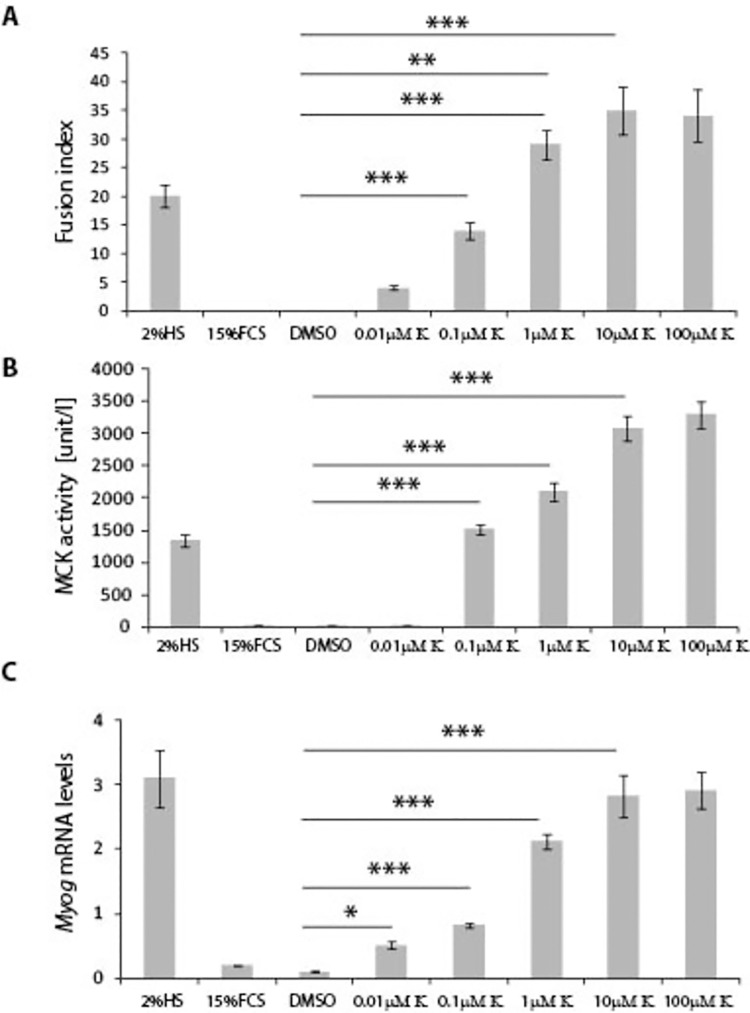
Effect of Kinetin on C2C12 myoblast differentiation in the mitogen enriched medium. **A)** Fusion index of C2C12 cells increased significantly in the presence of Kinetin at the 0.1μM, 1μM, 10μM and 100μM concentrations, in comparison to cells grown in the presence of GM and DMSO. **B)** MCK activity and **C)** *Myog* transcript levels were significantly higher in the presence of Kinetin at the 0.1μM, 1μM, 10μM and 100μM concentrations, in comparison to cells grown in the presence of GM and DMSO. Error bars are ± SEM (n = 6). One-way Anova with Bonferroni *post hoc* test: *p<0.05, **p<0.01, ***p<0.001. Scale bar 100 μm. FCS (Foetal Calf Serum); DM (Differentiation medium); GM (Growth medium); K (Kinetin); BA (N6-benzyladenine); MCK (Muscle Creatine Kinase); *Myog* (Myogenin).

Next, we investigated the concentration-dependent effect of Kinetin on C2C12 differentiation; C2C12 cells were grown in the GM supplemented with 0.01μM, 0.1μM, 1μM, 10μM and 100μM Kinetin for 7 days. Kinetin enhanced significantly fusion index (Fig 3A) at concentrations ranging from 0.1μM to 100μM. We also used quantitative assays and found that Kinetin had a significant effect on MCK activity (Fig 3B), at concentrations from 0.1μM to 100μM, as well increased *Myog* transcript levels (Fig 3C), at concentrations from 0.01μM to 100μM. It has to be noted that there was no significant difference in all 3 measurements between 10μM and 100μM Kinetin concentrations. Taken together, this might indicate that Kinetin consistently enhances myoblast differentiation between 1μM and 100μM concentration.

**Fig 4:**
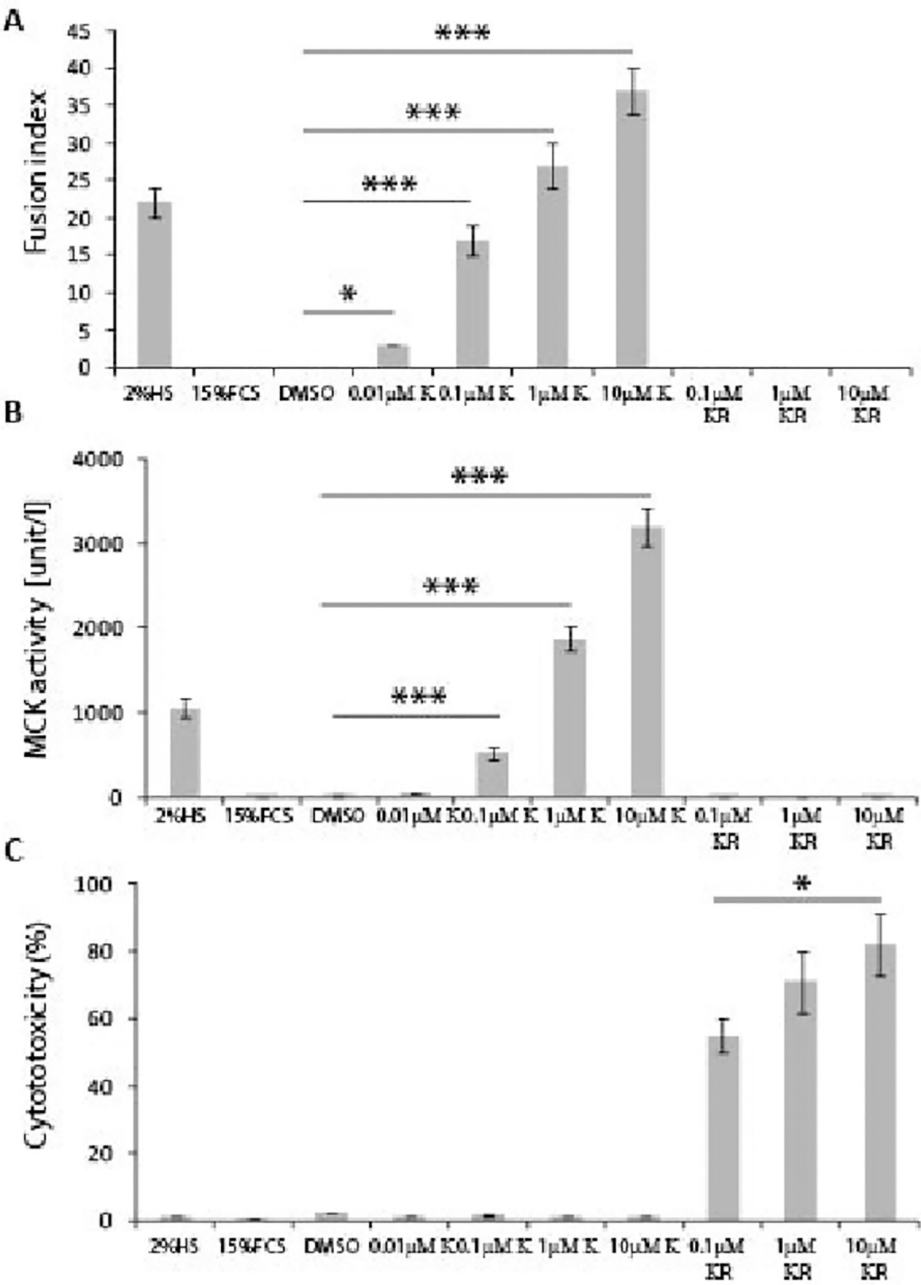
Kinetin riboside but not Kinetin is toxic in C2C12 cells. Kinetin but not Kinetin riboside supplement increased significantly **A)** fusion index and **B)** MCK activity, in C2C12 cells grown in GM with 15% FCS, in comparison to C2C12 myoblasts supplemented with DMSO only for 7 days. **C)** An MTT assay showed that Kinetin riboside but not Kinetin was toxic in C2C12 cells, at the tested concentrations, after 3 days in culture. Error bars are ± SEM (n = 6). One-way Anova with Bonferroni *post hoc* test: *p<0.05, **p<0.01, ***p<0.001. FCS (Foetal Calf Serum); DM (Differentiation medium); K (Kinetin); KR (Kinetin riboside); GM (Growth Medium); MCK (Muscle Creatine Kinase).

Since Kinetin riboside (KR) has been shown to display biological activities in mammalian cells, we tested its ability to induce myoblast differentiation in comparison with Kinetin. We found that KR did not stimulate C2C12 differentiation in the GM after 7 days in culture at either 0.1μM, 1μM or 10μM concentrations, as Fusion index was not changed (Fig 4A) and there was no enhanced MCK activity (Fig 4B). However, in this set of experiments, we observed that KR–but not Kinetin–might be toxic to C2C12 myoblasts. Hence, we performed an MTT assay to validate our observation and we found that KR was indeed toxic at 0.1μM, 1μM and 10μM concentrations (Fig 4C), while Kinetin did not show any toxicity at 0.01μM, 0.1μM, 1μM and 10μM concentrations, after 3 days in culture. We also observed no toxicity after supplementation of the GM with DMSO (Fig 4C). The cytotoxicity of KR was found to be dose-dependent as there was a significant difference in the cell viability at the 0.1μM and 10μM concentrations.

**Fig 5:**
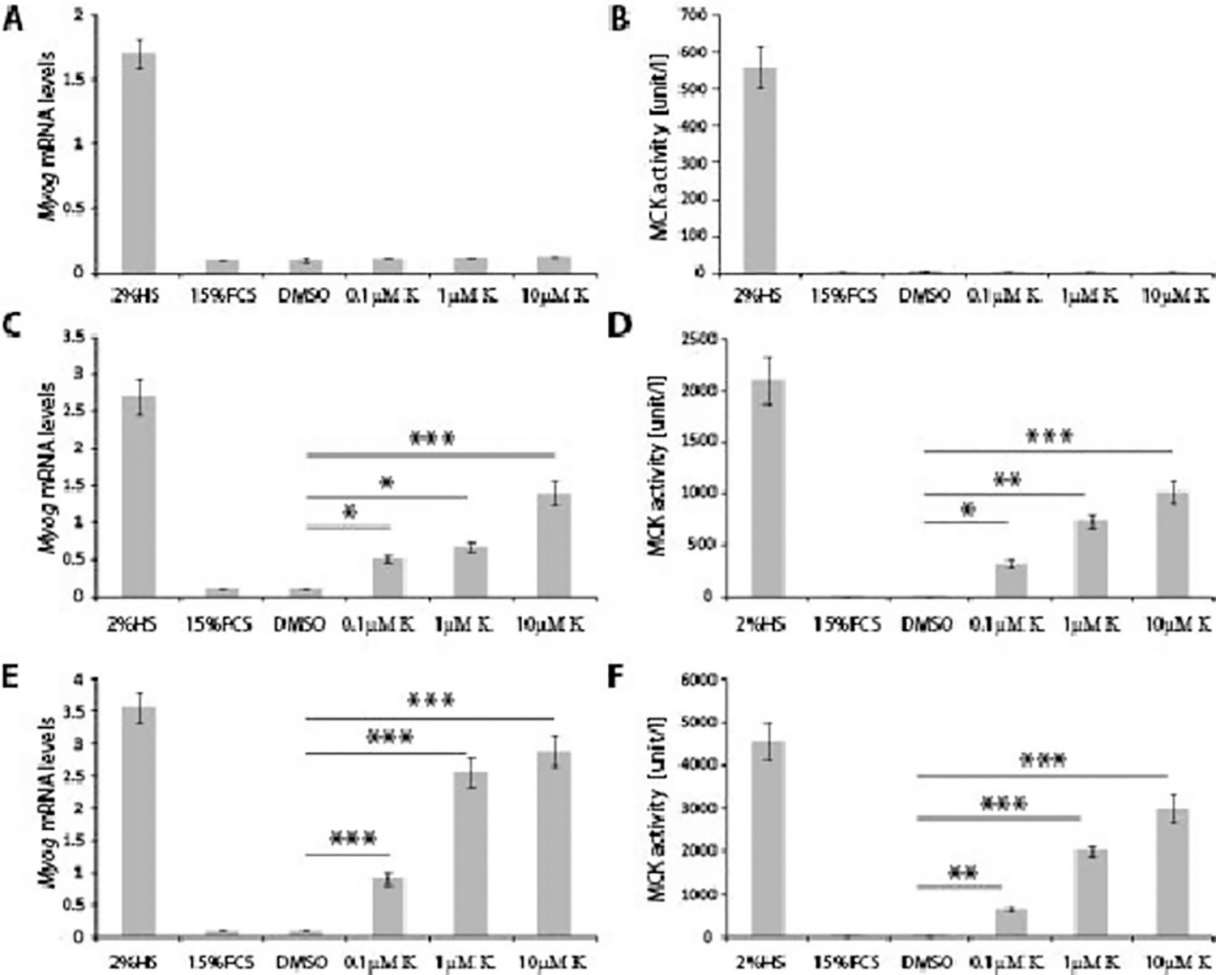
Time-dependent effect of Kinetin on C2C12 differentiation into myotubes. C2C12 cells were grown in GM supplemented with DMSO (negative control) or with different concentrations of Kinetin at 0.1μM, 1μM and 10μM. *Myog* transcript levels and MCK activity was validated at 3 days **(A and B)**, 5 days **(C and D)** and 7 days **(E and F)** post seeding C2C12 myoblasts. Error bars are ± SEM (n = 6). One-way Anova with Bonferroni *post hoc* test: *p<0.05, **p<0.01, ***p<0.001. FCS (Foetal Calf Serum); HS (Horse Serum); DM (Differentiation medium); GM (Growth medium);K (Kinetin); MCK (Muscle Creatine Kinase). *Myog* (Myogenin).

Next, we investigated time-dependent effects of Kinetin on C2C12 differentiation in GM. As described above, a significant stimulation of myoblast differentiation was observed after 7 days in culture, hence we performed a number of parallel cultures and assayed for effects of Kinetin after 3, 5 and 7 days. We found that Kinetin did not induce MCK activity or increase *Myog* mRNA levels after 3 days in culture (Fig 5A and 5B). However, there was a significant stimulation of differentiation of myoblasts after 5 days in culture (Fig 5C and 5D) based on both increased MCK activity and *Myog* transcript levels, at 0.1μM, 1 μM and 10μM concentrations. Subsequently, there was a significant effect of Kinetin after 7 days in culture in the GM supplemented with 0.1μM, 1μM and 10μM Kinetin, in comparison to C2C12 cells treated with DMSO, based on both MCK activity and *Myog* mRNA levels (Fig 5E and 5F). This indicates that Kinetin efficiently stimulates myoblast differentiation in the growth medium within 5 days in culture.

**Fig 6:**
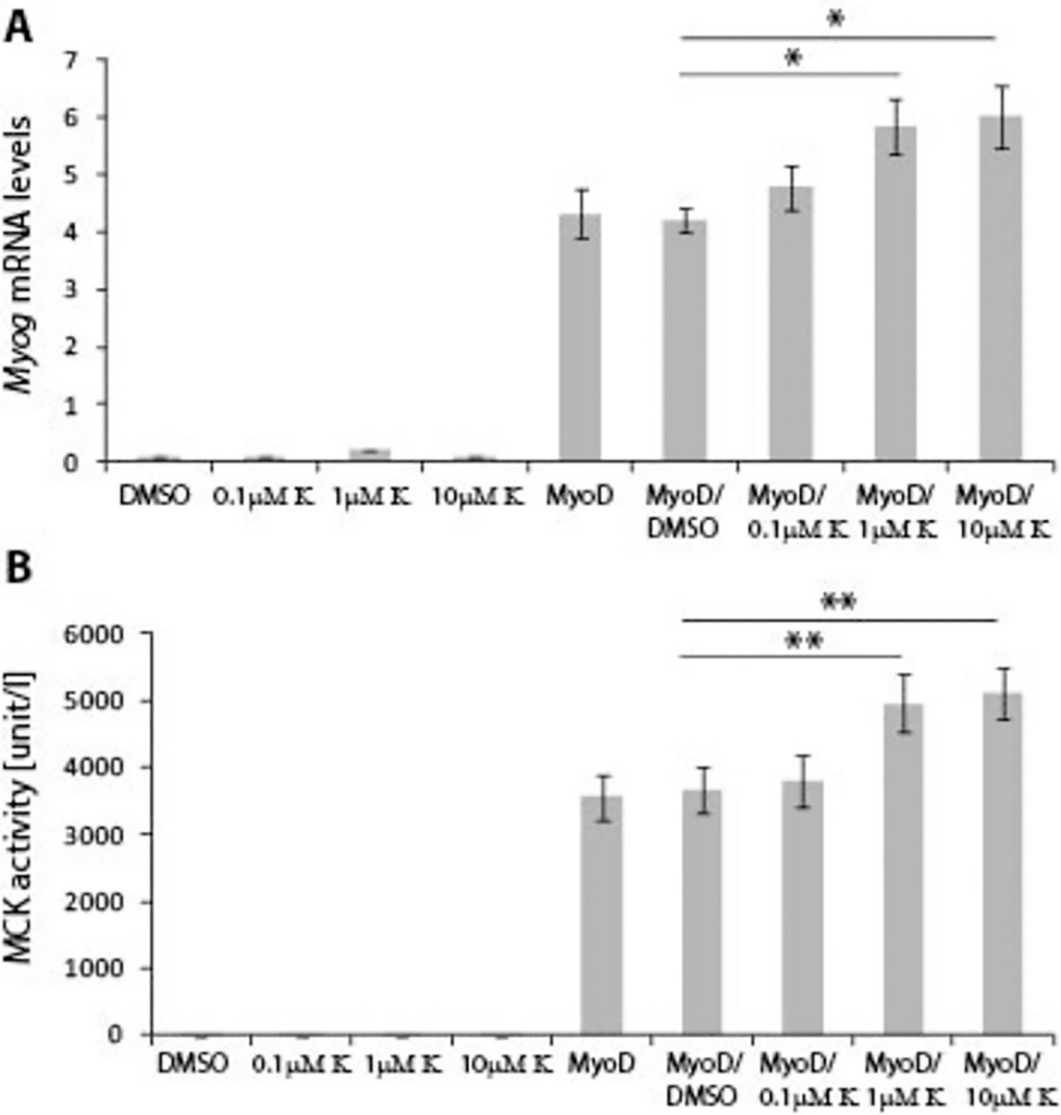
Kinetin enhances MYOD-dependent conversion of 10T1/2 fibroblasts into myotubes. 10T1/2 fibroblasts were transiently transfected with a full-length MyoD gene expression construct and were grown in DM supplemented with 5% horse serum. 24 hours post transfection, cells were supplemented with either DMSO (negative control) or Kinetin at 0.1μM, 1μM or 10μM concentration, and were grown for 6 days. Kinetin supplemented at the 1μM and 10μM concentrations significantly increased **A)** *Myog* transcript levels and **B)** MCK activity, which correlates with an increased conversion of fibroblasts into myotubes. Error bars are ± SEM (n = 8). One-way Anova with Bonferroni *post hoc* test: *p<0.05, **p<0.01, ***p<0.001. K (Kinetin); MCK (Muscle Creatine Kinase); *Myog* (Myogenin); DM–Differentiation Medium.

Finally, we assessed the ability of Kinetin to potentiate MyoD-dependent conversion of murine embryonic fibroblasts into myotubes, as previously described [24]. It is well-established that embryonic fibroblasts, like the 10T1/2 cell line, can be differentiated into myotubes upon MyoD expression, in a so called conversion assay, for example in the differentiation medium (5%HS). Hence, we transiently transfected 10T1/2 cells with a full-length MyoD expression construct. At 24 hours post transfection, we replaced the GM with mitogen-poor medium (5%HS), supplemented with either DMSO or different concentrations of Kinetin (at 0.1μM, 1μM, 10μM concentrations). We assayed the effect of Kinetin on the conversion of fibroblasts into myotubes after 6 days in culture, using two objective quantitative assays. We found that Kinetin increased *Myog* transcript levels at 1μM and 10μM concentrations (Fig 6A), while 0.1μM Kinetin did not show a significant increase. MCK activity was also significantly stimulated at 1μM and 10μM concentrations of Kinetin only (Fig 6B). We also found that Kinetin by itself, without MyoD expression, was not able to stimulate conversion of 10T1/2 fibroblasts into myotubes: there was no increase of either MCK activity or *Myog* mRNA levels (Fig 6). In addition, Kinetin did not enhance MyoD expression from the plasmid used for the transient transfection (S2 Fig).

In order to explore a potential mechanism for Kinetin stimulation of differentiation of C2C12 myoblasts, we evaluated the potential efficacy of Kinetin to scavenge ROS. We found that Kinetin (at 1μM, 10μM, 10μM concentrations) significantly reduced ROS levels after 12h and 24h incubation (S3 Fig). Overall, our study indicates that Kinetin is a potent small molecule that efficiently stimulates the differentiation of myoblasts into myotubes. N6-benzyladenine was also able to stimulate myoblast differentiation, however with much lower efficacy and only in GM growth medium, while trans-Zeatin did not stimulate this process at all. It appears that the highest efficacy of Kinetin was reached between 1μM and 10μM concentrations, when Kinetin was supplemented into both the growth and differentiation media. We also found that Kinetin riboside but not Kinetin itself showed toxicity in C2C12 myoblasts. More importantly, Kinetin was also able to stimulate MyoD-dependent conversion of fibroblasts into myotubes. Taken together, our data clearly indicate that Kinetin should be recognised as a small molecule with potential for promoting muscle regeneration.

## Discussion

Although cytokinins were initially discovered as potent plant hormones (reviewed in [1]), evidence has been mounting over the past 20 years over their abilities to alter biochemical processes in mammalian cells, both *in vitro* and *in vivo*. More importantly, it has also been shown that one of the cytokinin family members, Kinetin, is found in DNA extracts from human fibroblasts [28] and in human urine from cancer patients [14]. The mechanism by which Kinetin is being formed in human DNA is based on the reaction of the amino group of adenine with the aldehyde group of furfural [29], which is itself formed as primary product of oxidative DNA damage [30]. However, probably the most spectacular features of Kinetin discovered so far, including in mammalian cells, are its anti-aging properties. The latter have been shown in fruit flies [8], *Caenorhabditis elegans* [10] and in human mammary skin fibroblasts [11]. Furthermore, a number of studies showed that Kinetin can also regulate several critical biological processes in mammalian cells including calcium signalling and Reactive Oxygen Species (ROS) homeostasis (reviewed in [31]). Since these processes have been shown to be important during muscle regeneration (reviewed in [32]), in this study we sought to investigate the pro-myogenic effects of Kinetin and other two small molecules, N6-benzyladenine and trans-Zeatin, belonging also to the cytokinin family of plant hormones. Specifically, we employed an *in vitro* model of myoblast differentiation into myotubes. C2C12 cells, derived from murine skeletal muscle cells, are a well-established model to study muscle regeneration and differentiation *in vitro* [33]. During differentiation of C2C12 cells, myoblasts undergo a complex remodelling to form mature myotubes in parallel with the increased expression of a number of muscle-specific genes [34, 35]. In our study, in order to monitor the differentiation process in quantitative way, we chose to assess the impact of cytokinins based on Fusion index, the level of Myogenin (based on Taq-man qPCR assay) and Muscle Creatine Kinase (MCK) activity. It has been shown that *Myog* transcript levels increase steadily during differentiation of myoblasts into myotubes [36], and *Myog* knock-down leads to defective terminal muscle differentiation [37]. The other quantitative assay used in this report was based on MCK activity increases during C2C12 differentiation [26].

We found that Kinetin, at 1μM and 10μM concentrations, stimulates myoblast differentiation in both mitogen-rich and mitogen-poor media with the highest efficacy. The concentration of Kinetin established in our study is in line with previous studies in different mammalian cell lines, where it has been reported that Kinetin shows biological activities at μM concentrations (reviewed in [31]). N6-benzyladenine also significantly enhances formation of myotubes, however we found that this effect was less potent than that of Kinetin and was restricted to the mitogen-rich medium. Finally, we found that Zeatin did not affect myoblast differentiation. This could be explained by a potential mechanism—yet to be discovered—in which Kinetin, but not other cytokinins, stimulate myoblast differentiation *in vitro*. Firstly, it has been shown that muscle differentiation is regulated by calcium signals. In fact, the expression of Myogenin requires the activation of CaMKII and the rise of intracellular Ca2+ levels is a prerequisite for the activation of the fusion process of myoblasts [38]. Kinetin and Zeatin, but not N6-benzyladenine, were found to stimulate Ca2+ influx in plants and induced bud formation, likely through voltage-dependent DHP-sensitive Ca2+ channels on the plasma membrane [39]. Interestingly, Kinetin treatment led to increased expression of various differentiation markers in keratinocytes exposed to high Ca2+ levels [40]. Furthermore, the increase of intracellular calcium levels is responsible for ROS formation through the action of the mitochondrial respiratory chain, which plays a critical role during myogenic differentiation [41]. In fact, treatment of C2C12 cells with a ROS-trapping agent (phenyl-N-tert-butylnitrone, PBN) enhanced myoblasts differentiation, while addition of 25μM H_2_O_2_ to cells in 20% O_2_ dramatically slowed differentiation down and lowered *Myog* transcript levels [19]. Perhaps crucially, Kinetin, but not other cytokinins, was reported as a potent ROS scavenger. Using a biochemical assay, Kinetin has been reported to protect DNA against Fenton reaction-mediated oxidative damage and significantly reduced formation of 8-hydroxy-2-deoxyguanosine formation, which is frequently used as a marker of oxidative DNA damage [13]. Furthermore, Kinetin was shown to be neuroprotective in the HT22 cellular model of glutamate-induced oxidative toxicity. This neuroprotection was due to suppression of intracellular ROS accumulation and increases of intracellular calcium influx [16]. Similarly, we found that Kinetin lower ROS levels in the C2C12 myoblasts and this could be one of many potential mechanisms that Kinetin utilizes to stimulate muscle differentiation *in vitro*. We also found that the Kinetin derivative Kinetin riboside is toxic in C2C12 myoblasts. It should be noted that a similar effect of Kinetin riboside—but not Kinetin—has been previously reported in a number of cancer cell lines. Kinetin riboside induces cell death and attenuates G1 to S transition in HepG2 cells [42]. Moreover, Kinetin riboside induces apoptosis in HeLa and mouse melanoma B16F-10 cells, through a mitochondrion-dependent apoptosis pathway [43]. Furthermore, Kinetin riboside inhibited proliferation in HCT-15 human colon cancer cells, in a dose-dependent manner [44].

We also wanted to know whether supplying Kinetin enhances the recruitment of non-muscle cells into the myogenic fate. This hypothesis was based on the assumption that the presence of additional key regulatory factors might facilitate MyoD-mediated initiation of regulatory circuits that drive myogenic differentiation. It is well-established that myogenic factors like MyoD are able to reprogram numerous cell types, including fibroblasts, so that they adopt a muscle fate [45]. We found that Kinetin significantly enhanced MyoD-dependent conversion of fibroblasts into myotubes. However, Kinetin alone did not give rise to such conversion. This clearly demonstrates that Kinetin supplements the molecular repertoire of various key regulatory factors to facilitate MyoD-mediated myogenic differentiation.

## Conclusions

Overall, our current study identified a novel biological activity of Kinetin in mammalian cells. For the first time, we showed that Kinetin at micromolar concentrations robustly stimulates C2C12 myoblast differentiation, in both mitogen-rich and mitogen-poor conditions. This may potentially be achieved via a mechanism related to the ability of Kinetin to alter intracellular calcium levels, as well its biological activity in ROS regulation. In addition, Kinetin proved to be a potent enhancer of MyoD-dependent conversion of fibroblasts into myotubes. Taken together, our findings suggest the existence of a new class of small molecules with potent pro-myogenic activities. In the future, this may open new therapeutic avenues for diseases of skeletal muscle degeneration and potentially in age-related muscle wasting.

## Supporting information

S1 Fig Experimental workflow. Study time-frame for C2C12 myoblasts grown in **(A)** growth media [related to Figs 1, 3–5], **(B)** differentiation media [related to Fig 2] and 10T1/2 fibroblasts conversion into myotubes assay [related to Fig 6] **(C)**. GM (Grown Media); DM (Differentiation Media).
(TIF)

S2 Fig Kinetin does not enhance MyoD expression in 10T1/2 fibroblasts. 10T1/2 fibroblasts were transiently transfected with a full-length MyoD gene expression construct and were grown in DM supplemented with 5% horse serum. 24 hours post transfection, cells were supplemented with either DMSO (negative control) or Kinetin at 0.1μM, 1μM or 10μM concentration, and were grown for 24H. Kinetin supplemented at the 1μM and 10μM concentrations did not increase MyoD transcript levels. Error bars are ± SEM (n = 6).
(TIF)

S3 Fig Kinetin efficiently reduces ROS levels in C2C12 myoblasts. C2C12 cells were grown in GM supplemented with DMSO (negative control) or with different concentrations of Kinetin at 1μM, 10μM and 10μM. ROS levels were assessed using a DCFDA / H2DCFDA fluorescent assay after **(A)** 12h and **(B)** 24h. Error bars are ± SEM (n = 10). One-way Anova with Bonferroni *post hoc* test: ***p<0.001. ROS (Reactive Oxygen Species); GM (Growth medium);K (Kinetin).
(TIF)
