# Supplementary figures and images for: Kinetin stimulates differentiation of C2C12 myoblasts
Source: PLoS One. 2021 Oct 13;16(10):e0258419. doi: 10.1371/journal.pone.0258419 (PMC8513909; doi:10.1371/journal.pone.0258419)

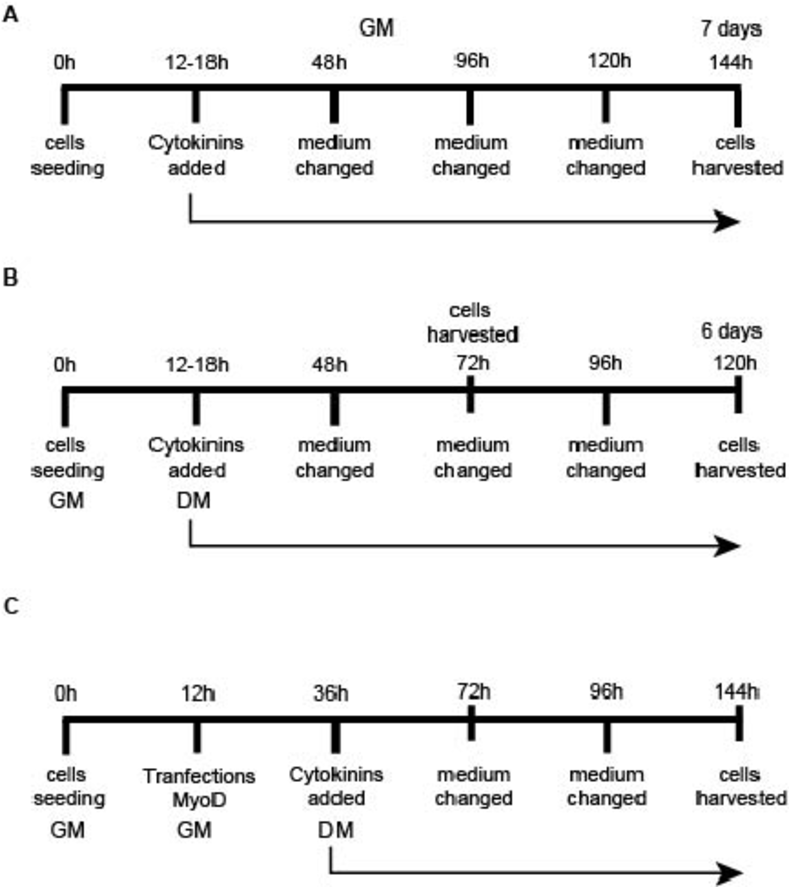

Supplement: S1 Fig — Study time-frame for C2C12 myoblasts grown in (A) growth media [related to Figs 1, 3–5], (B) differentiation media [related to Fig 2] and 10T1/2 fibroblasts conversion into myotubes assay [related to Fig 6] (C). GM (Grown Media); DM (Differentiation Media). (TIF) [file pone.0258419.s001.tif]

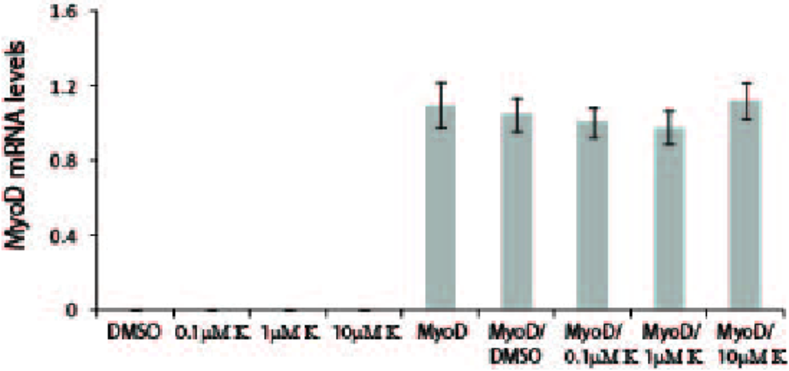

Supplement: S2 Fig — 10T1/2 fibroblasts were transiently transfected with a full-length MyoD gene expression construct and were grown in DM supplemented with 5% horse serum. 24 hours post transfection, cells were supplemented with either DMSO (negative control) or Kinetin at 0.1μM, 1μM or 10μM concentration, and were grown for 24H. Kinetin supplemented at the 1μM and 10μM concentrations did not increase MyoD transcript levels. Error bars are ± SEM (n = 6). (TIF) [file pone.0258419.s002.tif]

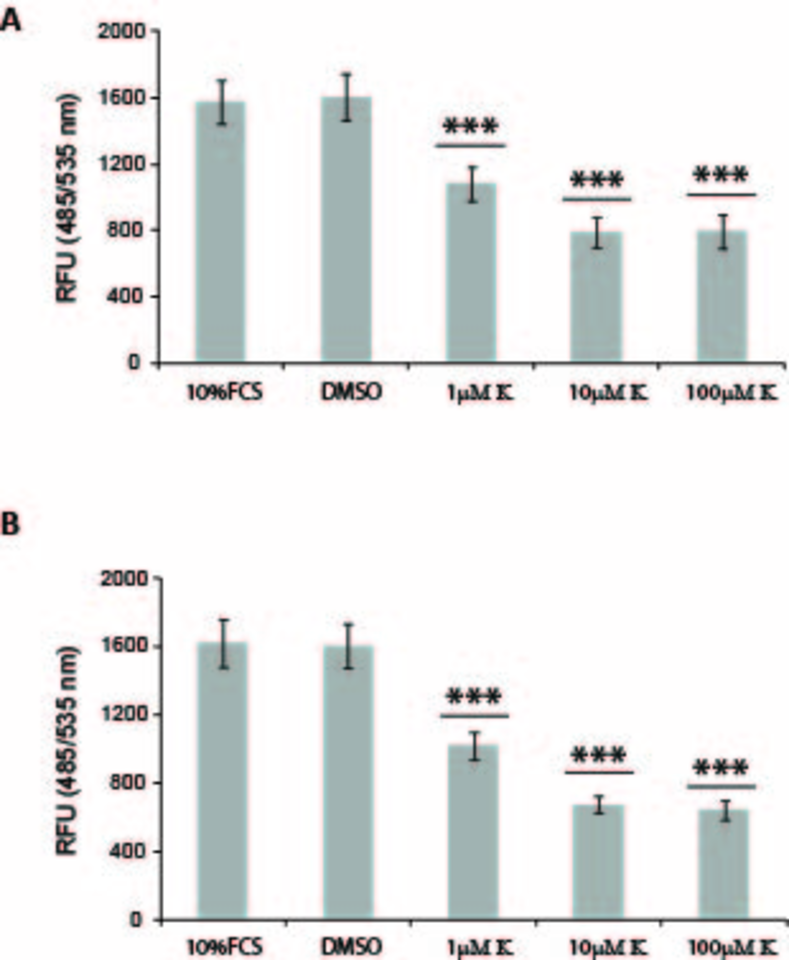

Supplement: S3 Fig — C2C12 cells were grown in GM supplemented with DMSO (negative control) or with different concentrations of Kinetin at 1μM, 10μM and 10μM. ROS levels were assessed using a DCFDA / H2DCFDA fluorescent assay after (A) 12h and (B) 24h. Error bars are ± SEM (n = 10). One-way Anova with Bonferroni post hoc test: ***p<0.001. ROS (Reactive Oxygen Species); GM (Growth medium);K (Kinetin). (TIF) [file pone.0258419.s003.tif]
